# In-Process Ultrasonic Monitoring and Nugget Geometry Evaluation in Resistance Spot Welding of Stainless Steel

**DOI:** 10.3390/ma19184001

**Published:** 2026-09-20

**Authors:** Jianan Lv, Xiaopeng Gu, Juan Dong, Guocheng Xu, Xiaoyan Gu, Jinjiang Li, Jialiang Hao

**Affiliations:** Key Laboratory of Automobile Materials of Ministry of Education, College of Materials Science and Engineering, Jilin University, Changchun 130025, China; lvjn24@mails.jlu.edu.cn (J.L.);

**Keywords:** resistance spot welding, in-process ultrasonic monitoring, A-scan signal, M-type ultrasonic image, random forest

## Abstract

Post-weld evaluation does not readily capture the dynamic evolution of nugget formation in resistance spot welding. In this study, an embedded ultrasonic probe was used to acquire A-scan signals during resistance spot welding of stainless steel. Consecutive signals were assembled into M-type ultrasonic images to analyze echo changes across the thermal cycle and under different heat inputs. Using 12 specimens with metallographic measurements of both nugget diameter D and thickness H, five- and three-dimensional acoustic features were extracted for D and H prediction, respectively. Random forest (RF) regression models were established and evaluated using leave-one-out cross-validation (LOOCV). The M-type images showed the streak evolution of interface echoes, back-wall echoes, and nugget-related echoes, reflecting differences in acoustic responses across welding stages and nugget states. The coefficient of determination (R^2^), mean absolute error (MAE), and root mean square error (RMSE) were 0.588, 0.674 mm, and 0.798 mm for D, respectively, with corresponding values of 0.570, 0.150 mm, and 0.198 mm for H. These preliminary results indicate quantitative correlations between the extracted low-dimensional acoustic features and nugget geometry, providing an experimental basis for nugget geometry evaluation using in-process ultrasound.

## 1. Introduction

Resistance spot welding is widely used in the manufacture of stainless-steel car bodies, automotive structural components, and thin-sheet lap structures. The combined action of current-induced heating and mechanical pressure causes temperature changes, plastic deformation, and interfacial contact evolution within the joint, requiring welding quality to be evaluated through both nondestructive testing and process monitoring [1,2]. Nugget diameter D characterizes the lateral bonding range and is an important geometric parameter for joint quality evaluation. Its relationship with joint load-bearing capacity has been investigated experimentally [3]. Nugget thickness H describes the extent of fusion in the through-thickness direction and, together with D, characterizes nugget geometry. Post-weld inspection focuses on the final bonding state, whereas in-process monitoring can provide additional dynamic information during nugget formation and solidification.

The amplitude, arrival time, and waveform changes in ultrasonic echoes are closely related to the acoustic properties and internal bonding state of a joint. Hua et al. used an ultrasonic probe embedded in the electrode for in situ post-weld inspection to calculate nugget size and classify welding states [4]. Yang et al. obtained spatial scan information on spot welds using spiral C-scan and further combined this approach with deep learning for signal classification and nugget geometry measurement [5,6]. Non-contact laser ultrasound has also been used for post-weld diameter measurement. Hu et al. calculated nugget diameter from variations in the power spectral density of Lamb waves [7], while Nomura et al. estimated diameter from the arrival time of diffraction waves and their propagation velocity [8]. These studies represent different approaches to post-weld ultrasonic geometry evaluation. For in-process monitoring, Liu et al. continuously acquired ultrasonic signals with an embedded probe to track nugget growth and solidification and perform online diameter measurement [9], indicating the potential of process echoes for nugget-geometry evaluation.

Arranging consecutive A-scan signals in acquisition order produces M-type images containing both time of flight (TOF) and process-time information. Andreoli et al. arranged consecutive A-scan signals from resistance spot welding into B-scans with time of flight and welding time as the two axes. They examined relationships between in-process ultrasonic parameters, including TOF, and nugget diameter measured by peel testing [10]. Scott et al. applied deep-learning semantic segmentation to M-scans acquired during spot welding to identify weld-pool and sheet-stack regions, extract dynamic weld penetration, and estimate final nugget diameter [11]. These studies provide a basis for using time-resolved images to analyze nugget evolution and geometry.

In other welding processes, Sweeney et al. constructed TOF plots with acquisition time on the horizontal axis from consecutive signals recorded by a stationary phased array to monitor weld-pool changes during multi-pass tungsten inert gas (TIG) welding [12]. Nomura et al. continuously acquired laser-ultrasonic B-scans with excitation position and time of flight as the axes to observe sound velocity changes and interface-echo transitions during lap TIG spot welding [13]. Dimakos et al. acquired phased array B-scans during robotic TIG welding and used the weakening or disappearance of sidewall echoes during melting and their recovery after solidification as indicators of fusion state [14]. Although their imaging coordinates and evaluation targets differ, these methods all use continuously acquired ultrasonic signals to characterize heating, melting, or solidification.

Studies combining ultrasonic features with data-driven methods cover both spot weld quality classification and size estimation. Ultrasonic time-frequency features have been used with support vector machines and tree-based models to classify spot-weld strength or quality [15,16]. Ghafarallahi et al. combined post-weld A-scan signals, acoustic simulation, and neural networks to estimate nugget diameter in three-sheet spot-welded joints and assess quality based on diameter [17]. Ulbrich and Kańczurzewska developed a multiple linear regression model for nugget diameter using ultrasonic echo parameters [3]. Studies on domain-knowledge-driven spot weld quality modeling and interpretable ultrasonic machine learning also inform feature selection and interpretation of results [18,19,20]. Building on existing post-weld diameter measurement, process-parameter correlation, and boundary segmentation methods, this study examines how process-echo changes in M-type images can be summarized as fixed low-dimensional acoustic features and quantitatively related to D and H separately, linking qualitative image observations to quantitative evaluation of nugget geometry.

This study uses an embedded ultrasonic probe to record in-process signals during resistance spot welding of stainless steel and analyzes echo evolution across thermal-cycle stages and heat inputs. Based on this analysis, five-dimensional and three-dimensional acoustic features are constructed for D and H, respectively, with explicit computational definitions and signal interpretations. Random forest regression and leave-one-out cross-validation are used to examine the quantitative relationships between these features and metallographically measured nugget geometry, providing experimental evidence for nugget-geometry evaluation based on in-process ultrasound.

## 2. Materials and Methods

### 2.1. Materials and Specimen Preparation

**Figure 1:**
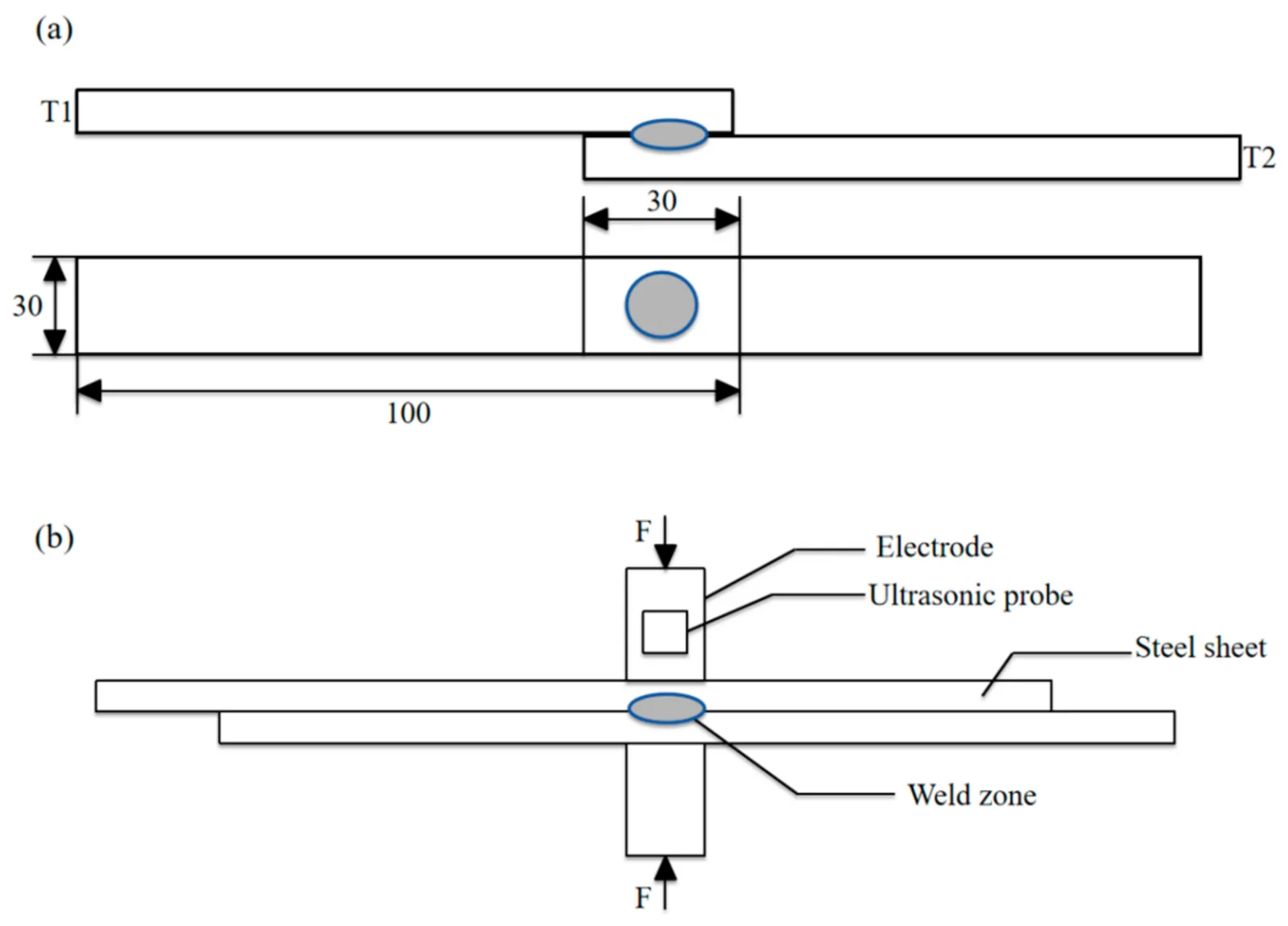
Geometry of the spot-welded lap-joint specimen and electrode arrangement with the embedded ultrasonic probe: (**a**) lap-joint specimen geometry and (**b**) electrode arrangement with the embedded ultrasonic probe.

Austenitic stainless steel sheets were used as the test material. This material is widely used in rail-vehicle car body structures because of its corrosion resistance and overall mechanical properties. The specimens had a two-sheet lap-joint configuration. The upper and lower sheets were both 1.5 mm thick, and each sheet had dimensions of 100 mm × 30 mm with a lap length of 30 mm. The specimen geometry and the electrode arrangement with the embedded ultrasonic probe are shown in Figure 1.

The main chemical composition and acoustic constants of stainless steel are listed in Table 1 and Table 2, respectively.

The resistance spot-welded specimens were prepared using a YR-500C AC resistance spot welding machine (Panasonic Welding Systems (Tangshan) Co., Ltd., Tangshan, China). Single-pulse and dual-pulse current schedules were adopted. For the single-pulse schedule, the welding current was 1.5–9 kA, and the current duration was 11, 15, or 20 cycles. For the dual-pulse schedule, the first current pulse was used for preheating at 2–5 kA for 10 cycles. The cooling interval between the pulses was 10 cycles. The second current pulse was used to form the weld nugget at 4–9.5 kA for 15 or 20 cycles. The electrode pressure ranged from 0.15 to 0.3 MPa. These are the overall ranges of the conditions used. Not all combinations of the listed parameters were tested. To obtain joints under different heat inputs, the pulse schedule, current, current duration, and electrode pressure were varied.

This study used 12 specimens with metallographic measurements of nugget diameter D and thickness H for subsequent quantitative analysis and regression modeling. After in situ ultrasonic acquisition during welding, the specimens were sectioned by wire cutting to obtain a through-thickness cross-section passing through the nugget center. The sections were prepared by mounting, grinding, polishing, and chemical etching in sequence. The sections were observed using an optical microscope, and spatial calibration was performed on the metallographic images. The nugget diameter D and thickness H were defined as the maximum transverse and through-thickness dimensions of the fusion zone, respectively. The heat-affected zone and plastic ring region were excluded from these geometric labels [21].

The welding conditions and corresponding specimen counts, including the subset used for quantitative D/H analysis, are summarized in Table S1 (Supplementary Materials).

### 2.2. Real-Time Ultrasonic Acquisition System and Method

**Figure 2:**
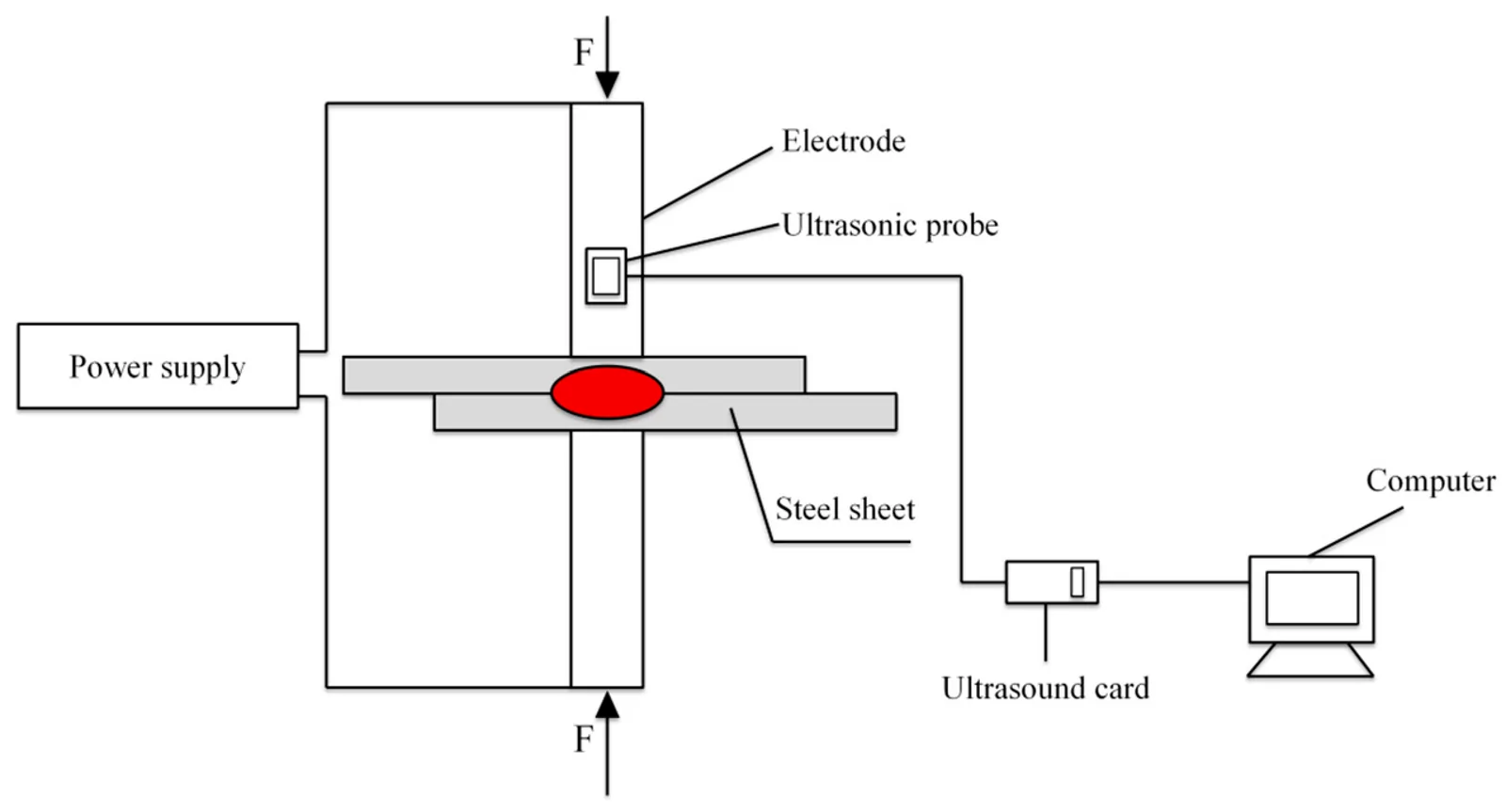
Schematic diagram of the ultrasonic signal acquisition system for resistance spot welding.

An embedded in-process ultrasonic sensing system was used to acquire real-time signals during weld nugget growth in resistance spot welding. The system consisted of the resistance spot welding equipment, an electrode shank with an embedded ultrasonic probe, a pulser/receiver module, a data acquisition card, and a computer, as shown in Figure 2. The ultrasonic probe had a center frequency of 10 MHz and an element diameter of 6 mm. The −6 dB bandwidth of the ultrasonic acquisition card was 18 MHz, the sampling frequency was 80 MHz, and the A/D resolution was 12 bits. The excitation voltage was 230 V, the pulse width was 3 μs, and the receiver gain was 40 dB.

During welding, the ultrasonic probe was fixed inside the electrode shank and continuously emitted pulsed longitudinal waves toward the welding region along the through-thickness direction while receiving echo signals. According to the propagation theory of ultrasonic waves at heterogeneous interfaces, reflection and transmission occur when the acoustic path encounters interfaces with acoustic impedance differences, such as the solid–liquid interface between the base metal and the weld nugget, the lap interface, and the back-wall boundary [22,23]. The amplitude, arrival time, and waveform characteristics of echoes in the A-scan signals are closely related to the acoustic properties and geometric dimensions of the nugget region. When consecutive A-scan signals acquired during the entire welding process are arranged in chronological order, an M-type ultrasonic image can be constructed. In this image, the vertical and horizontal axes represent the sampling-point index and acquisition sequence, respectively, thereby preserving interface-position information along the through-thickness direction and displaying the dynamic evolution of acoustic responses during nugget growth and solidification.

To quantitatively analyze the relationship between in-process ultrasonic responses and nugget geometry, complete time-series A-scan signals were extracted from specimens produced under different welding heat inputs. These signals were combined with metallographically measured D and H labels to establish a mapping dataset between in-process ultrasonic features and nugget geometric parameters.

### 2.3. Acoustic Feature Extraction and Model Evaluation

Acoustic features were extracted from the complete two-dimensional A-scan matrix of each specimen. Matrix row i represents the acquisition index, and column z represents the sampling-point index within an individual A-scan, which contains N_z_ sampling points. The complete matrix was used for feature calculation, rather than the cropped region used for image display.

The matrices were first linearly normalized using the 1st and 99.5th percentiles calculated for each matrix, and the values were clipped to [0, 1]. Total variation (TV) denoising using Chambolle’s algorithm was then applied along the sampling-point direction of each A-scan, with a weight of 0.03. The values were clipped to [0, 1] again to obtain matrix I. Row-wise median subtraction was applied to I to obtain Y. The amplitude envelope E was obtained from Y using the Hilbert transform along the sampling-point direction. E was averaged along the acquisition direction, followed by one-dimensional Gaussian smoothing with a standard deviation of max(2, N_z_/300) sampling points and boundary extension by replicating the nearest endpoint value. This gave the mean envelope curve p(z).

Four half-open windows were defined as fractions of N_z_: W_1_ = [0.02, 0.12), W_2_ = [0.12, 0.42), W_3_ = [0.42, 0.70), and W_4_ = [0.70, 0.98). The window boundaries were obtained by multiplying the corresponding fractions by N_z_ and converting the results to integer indices, with the upper boundary excluded. Let k_j_ denote the zero-based index of the peak of p(z) within window W_j_. Both peak positions and peak spacing were normalized by N_z_ − 1.

Table 3 defines the five features used for D prediction and the three features used for H prediction. P(f) denotes the mean squared magnitude, averaged along the acquisition direction, of the one-sided discrete Fourier transform of Y along the sampling-point direction. The zero-frequency component was set to zero, and the normalized frequency f ranged from 0 to 0.5 cycles per sampling point.

Here, b(i) = max_z∈W4_ E(i,z), μ_b_ and σ_b_ are the mean and population standard deviation of b(i) along the acquisition direction, respectively, and ε = 10^−8^. The mean calculations in Table 3 included all acquisition records and the sampling points specified for each feature. Amplitude and energy are relative signal quantities after normalization and denoising.

The feature combinations and some model parameters were determined using the available dataset. Feature selection considered random forest (RF) feature importance, SHapley Additive exPlanations (SHAP), correlations between features and labels, and prediction error. For H, the feature ranking also considered feature selection frequency across bootstrap resamples. The five D features and three H features listed in Table 3 were retained and held fixed in the subsequent evaluation.

Separate RF regression models were established with the metallographically measured D and H as the respective targets [24]. The D model used 150 trees with a maximum tree depth of 17. The minimum number of samples required to split an internal node was 4, and the minimum number of samples at a leaf node was 2. All five features were considered at each split. The H model used 200 trees with no limit on maximum tree depth. The minimum number of samples required to split an internal node was 4, and the minimum number of samples at a leaf node was 1. The number of features considered at each split was the square root of the total number of features. Both models used a random seed of 42, and no additional feature standardization was applied before model fitting.

Leave-one-out cross-validation (LOOCV) was used to evaluate predictions for the 12 specimens. In each fold, the model was trained on 11 specimens to predict the remaining specimen. An RF model was retrained in each fold using the fixed feature combinations and model parameters described above. The 12 out-of-fold predictions were pooled to calculate the coefficient of determination R^2^, mean absolute error (MAE), and root mean square error (RMSE). R^2^ was calculated once from all held-out predictions, not by averaging R^2^ values from individual single-specimen folds. Feature combinations and some parameters were determined using the available sample labels and prediction errors. This evaluation assesses the selected model rather than providing an independent validation of the entire model selection procedure.

## 3. Results and Discussion

### 3.1. Spot Welding Thermal Cycle and A-Scan Signal Analysis

**Figure 3:**
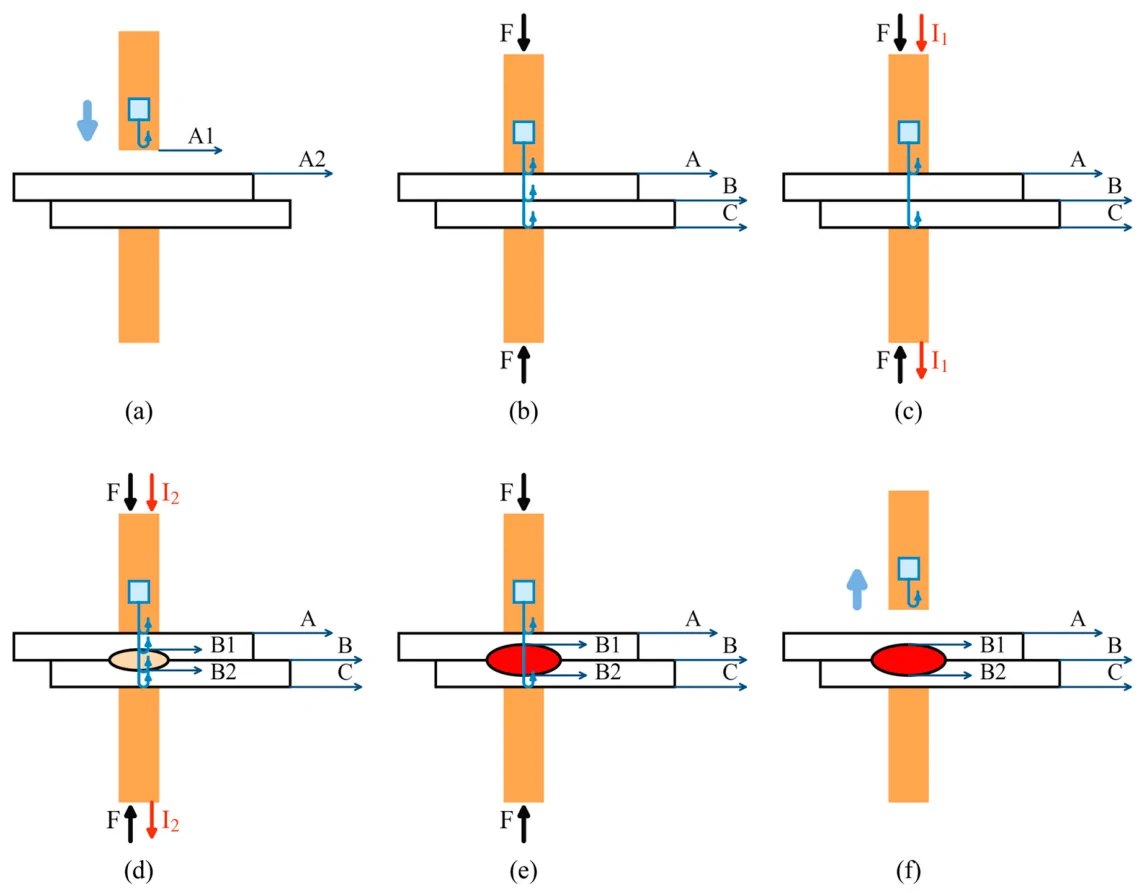
Spot welding thermal cycle and reflection-position schematic: (**a**) welding preparation, (**b**) pre-pressure, (**c**) pulse I and cooling, (**d**) pulse II, (**e**) cooling and holding, and (**f**) end of welding. Pale yellow depicts nugget formation, while red depicts the cooling and post-weld stages. Light-blue arrows indicate electrode movement, and curved blue arrows indicate ultrasonic reflections.

The dual-pulse spot welding process can be divided into six stages: welding preparation, pre-pressure, pulse I and cooling, pulse II, cooling and holding, and end of welding. Changes in electrode pressure, inter-sheet contact state, and temperature field alter the propagation path, sound velocity, and boundary reflection conditions of longitudinal ultrasonic waves traveling along the through-thickness direction [12,25]. The main reflection positions are denoted as A1 (electrode cap end face), A2 (upper surface of the upper sheet), B (contact interface between the upper and lower sheets), and C (lower surface of the lower sheet). After pre-pressure, A1 and A2 merge acoustically and appear as A. After nugget formation during pulse II, the original interface reflection at B weakens, and reflections mainly originate from the upper and lower nugget surfaces, denoted as B1 and B2. Figure 3 shows the thermal-cycle process and the reflection positions.

During welding preparation, the electrode has not yet applied stable pressure and stable acoustic coupling has not formed. During pre-pressure, the overlapped sheets gradually come into contact without current input. Pulse I produces preheating, followed by a short current interruption under maintained electrode pressure, during which the interfacial contact state and local temperature continue to change. During pulse II, the welding current causes a liquid weld nugget to form and grow in the inter-sheet region. The nugget then solidifies during cooling and holding, and the workpiece enters the post-weld state after pressure release.

**Figure 4:**
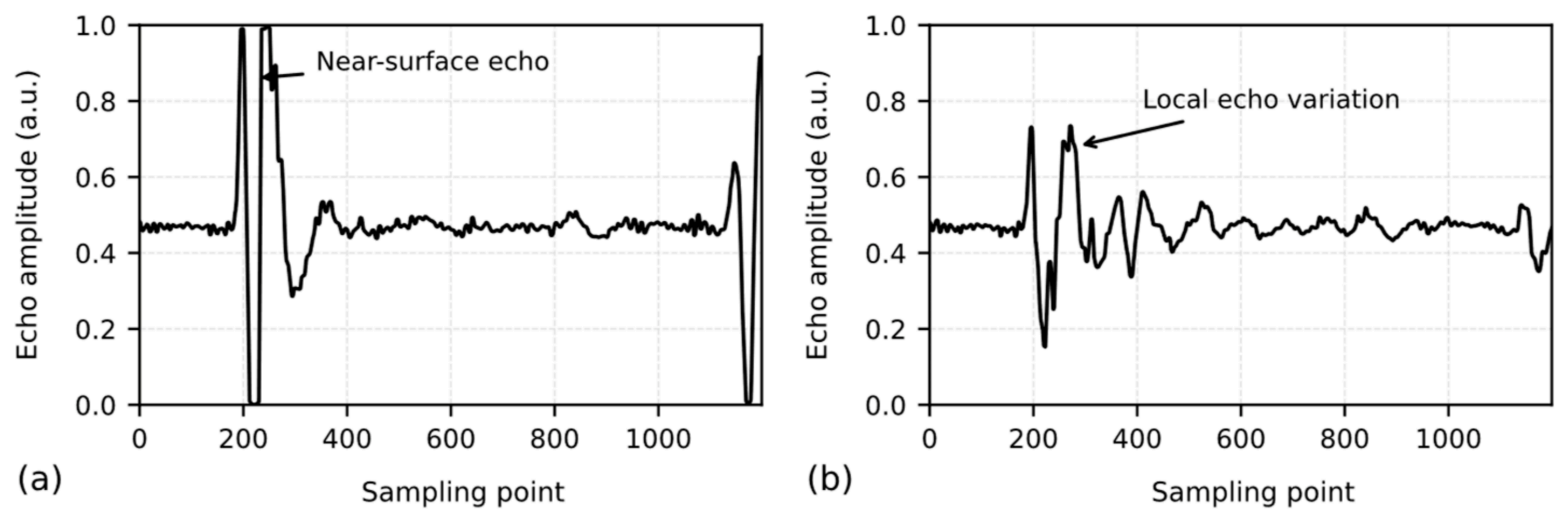
A-scan curves during welding preparation and pre-pressure: (**a**) welding preparation and (**b**) pre-pressure.

The welding preparation stage is shown in Figure 4a. Ultrasonic waves are mainly reflected at A1, the electrode cap end face, producing a strong near-surface peak in the A-scan signal, whereas reflections from A2, B, and C are essentially invisible. This signal mainly reflects the initial coupling condition and serves as a reference for subsequent echo changes.

Figure 4b corresponds to the pre-pressure stage. The A reflection becomes stable, and reflections from B, the inter-sheet interface, and C, the lower surface of the lower sheet, gradually increase and become distinguishable. Because no current is applied, these changes mainly indicate pressure-induced improvement in electrode-workpiece contact and inter-sheet coupling.

**Figure 5:**
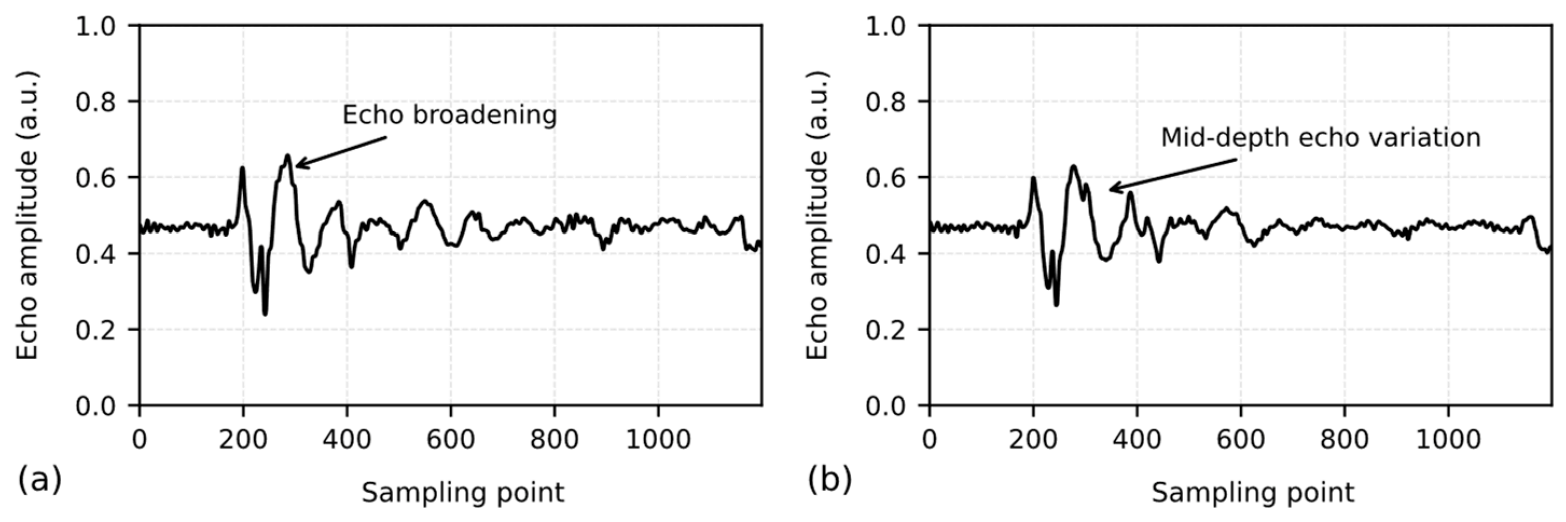
A-scan curves during pulse I and pulse II: (**a**) pulse I and cooling and (**b**) pulse II.

Figure 5a shows the pulse I and cooling stage. Resistance heating begins to affect the A-scan signal as the temperature rise reduces sound velocity, increases thermal attenuation, and, together with local plastic deformation and improved interfacial contact, changes ultrasonic propagation characteristics [23,25]. Reflections from A, B, and C shift slightly toward later arrival times, and the reflection peaks may weaken, broaden, or undergo local redistribution. At this stage, the B reflection mainly reflects the preheated inter-sheet contact state and does not yet represent an independent nugget-boundary response.

When the process enters pulse II, the inter-sheet region melts and the weld nugget begins to form and grow, as shown in Figure 5b. As fusion bonding develops, the original acoustic impedance discontinuity at B weakens, and the inter-sheet interface reflection decreases and is redistributed. Meanwhile, the upper nugget surface B1 and lower nugget surface B2 become new reflection positions, producing peak changes and waveform broadening in the middle region of the A-scan signal. The C reflection is also affected by transmitted energy and high-temperature attenuation, so its amplitude and peak shape vary with nugget growth.

**Figure 6:**
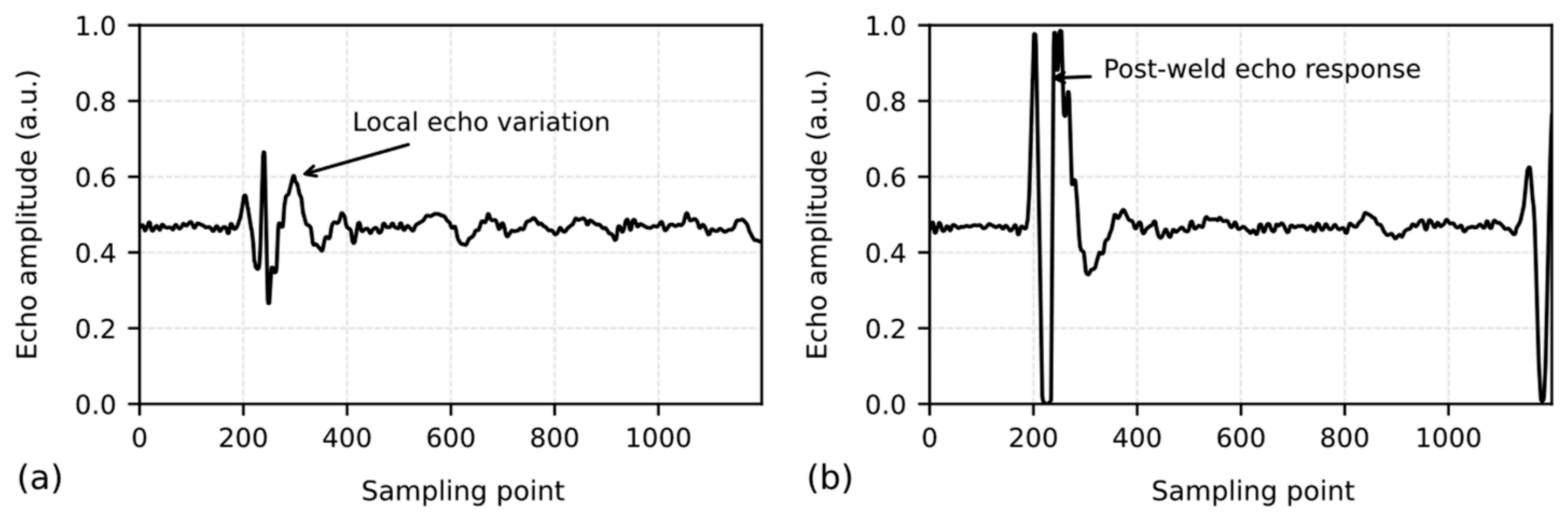
A-scan curves during cooling and holding and at the end of welding: (**a**) cooling and holding and (**b**) end of welding.

Figure 6a shows the cooling and holding stage. As the temperature decreases, sound velocity and ultrasonic attenuation gradually recover, and the B1/B2 reflections become stable during solidification. Compared with pulse II, fluctuations in peak position and amplitude decrease, indicating a weaker transient thermal effect. The original inter-sheet interface at B has transformed into a metallurgically bonded region, while recovery of the C reflection indicates that ultrasonic waves can again pass through the joint region more stably, although its amplitude and waveform remain affected by the solidified joint structure.

Figure 6b shows the end of welding. The A reflection differs from that during holding because the electrode-workpiece coupling condition changes. At this point, the weld nugget has solidified, and B1/B2 represent the upper and lower surfaces of the post-weld nugget. Because metallurgical bonding has formed between the sheets, B no longer appears as the clear mechanical contact interface observed during pre-pressure, while the C reflection represents the integrated propagation response through the post-weld joint.

These A-scan results show that changes in peak position and amplitude associated with A, B, B1, B2, and C are closely related to inter-sheet contact, nugget formation and growth, and solidification. In particular, weakening of the original B reflection and the appearance of B1/B2 reflections during pulse II can serve as key acoustic indicators of nugget formation. Stabilized reflections during cooling, holding, and the end stage reflect the integrated acoustic response of the solidified joint and provide the basis for linking M-type image features with nugget geometry.

### 3.2. Evolution of Process Echoes in M-Type Images

A single A-scan signal reflects only the through-thickness echo response at one acquisition time and cannot describe continuous echo migration, broadening, or bending during welding. Temperature fluctuation, pressure variation, and coupling-state changes also make a single peak value unstable as an indicator of nugget geometry. Consecutive A-scan signals were therefore assembled into M-type images to observe echo evolution along the process direction.

**Figure 7:**
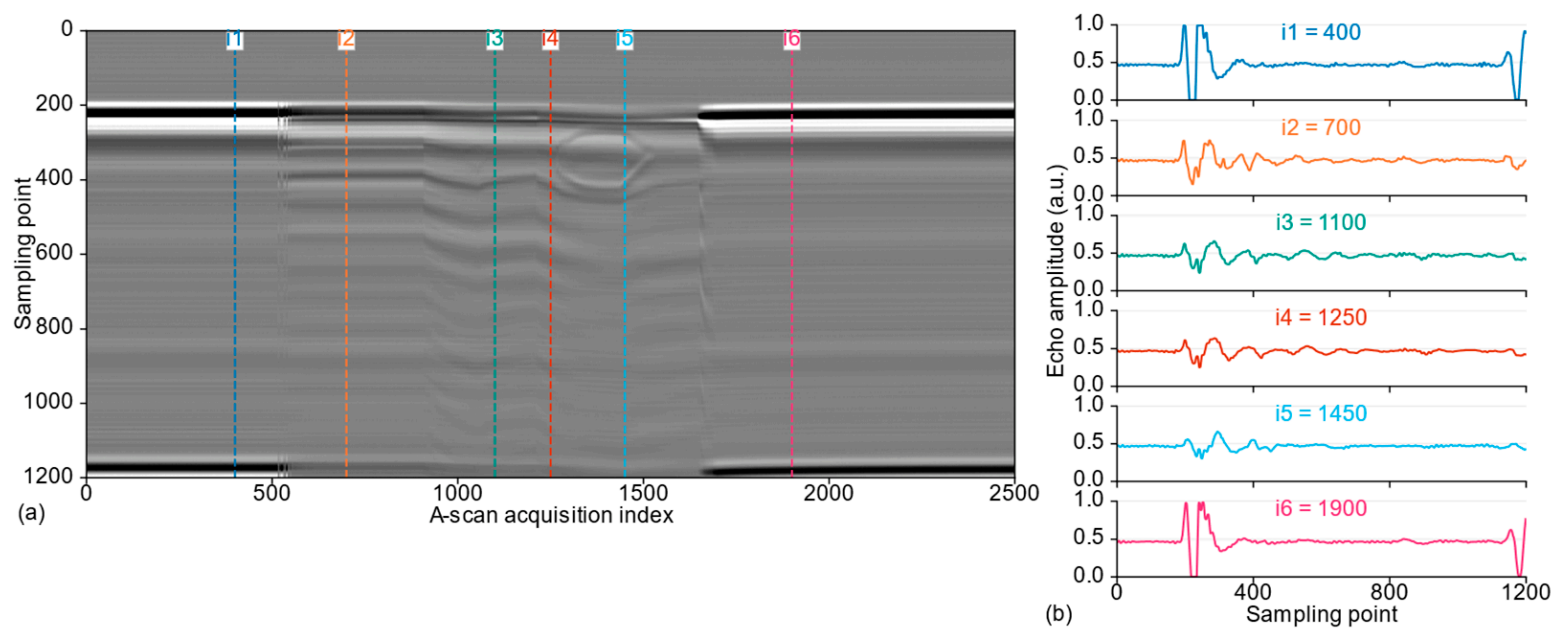
Construction of an M-type image from consecutive A-scan signals: (**a**) M-type image with selected acquisition indices and (**b**) corresponding A-scan traces.

To construct the M-type image, the two-dimensional A-scan matrix described in Section 2.3 was transposed for display. The horizontal axis represents the acquisition index, which can be converted to welding time, and the vertical axis represents the sampling-point index, which can be converted to propagation time or equivalent acoustic path length. The gray level represents echo amplitude, and each column is one A-scan signal [11]. Figure 7 shows the construction of an M-type image from consecutive A-scan signals.

**Figure 8:**
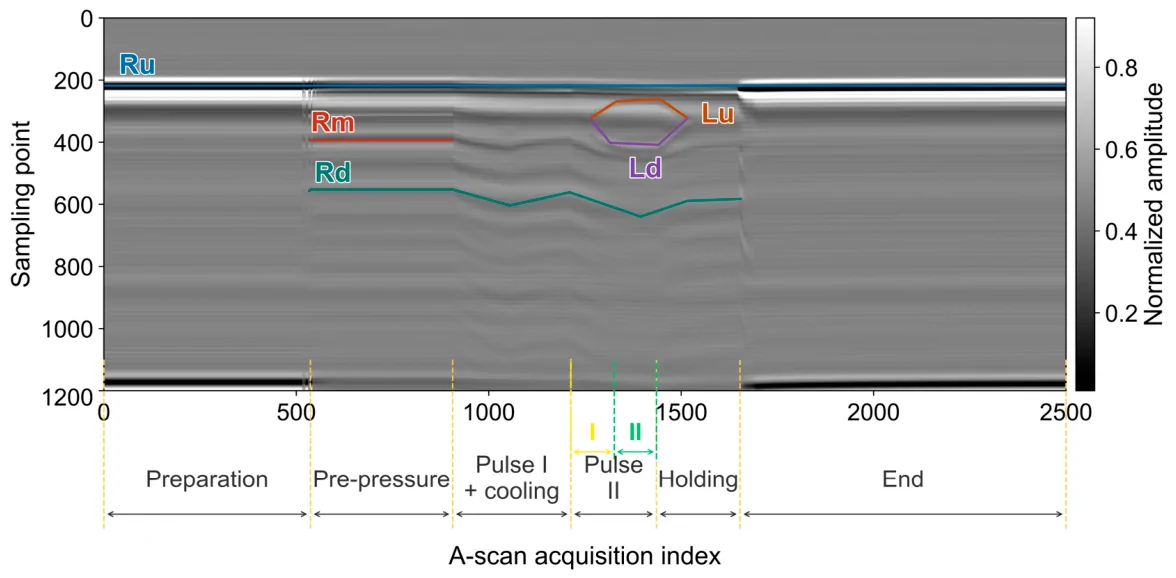
Representative M-type image and annotated main echo regions. I and II denote the nugget growth and relatively stable stages within pulse II, respectively.

In M-type images, changes in peak position, amplitude, and waveform across consecutive A-scan signals appear as streak migration, bending, broadening, or disappearance, making stage-dependent acoustic responses easier to identify. Figure 8 shows a representative M-type image and the annotated main echo regions. Ru, Rm, and Rd correspond to echoes associated with the electrode cap end face or upper surface, the inter-sheet interface, and the lower-sheet back wall, respectively. Lu and Ld correspond to echoes associated with the upper and lower solid–liquid interfaces of the weld nugget. The process direction is divided into six welding stages, with two Lu/Ld response stages further marked within pulse II.

M-type images emphasize the response of continuous echoes to physical transitions during welding rather than the localization of a single boundary. Other studies on ultrasonic monitoring during welding have also shown that received signals contain information related to melting and solidification [12].

The echo regions in the M-type image show changes related to joint state. Ru, the echo from the electrode cap end face or upper sheet surface, persists throughout welding as an approximately horizontal and stable bright streak and provides an acoustic path reference. Rm, the inter-sheet interface echo, is observed after pre-pressure and weakens or disappears after current heating as contact evolves, plastic deformation occurs, and metallurgical bonding develops. Rd, the lower-sheet back-wall echo, persists through multiple stages but bends toward a larger acoustic path during current application because temperature rise reduces sound velocity and increases echo arrival time; after current shutoff, it moves back as the temperature decreases [13]. The bending amplitude is related to heat input and can indirectly characterize thermal-cycle intensity.

Pulse II is the main stage of nugget formation and growth. After the second current pulse begins, heat input rapidly increases and the center of the inter-sheet region starts to melt. As the liquid nugget grows, the Lu/Ld spacing increases in the M-type image, indicating broadening of the nugget-related echo region, consistent with nugget growth in the through-thickness direction. The liquid nugget then approaches a relatively stable state, in which Lu/Ld spacing changes less and the two echoes appear nearly parallel. During cooling and holding, the current is stopped while pressure is maintained; the liquid region shrinks, the Lu/Ld spacing gradually decreases, and the nugget-related echo region narrows and gradually weakens. Thus, variation in Lu/Ld spacing can provide acoustic information on nugget evolution in the through-thickness direction.

These echo-region analyses show that Ru serves as a stable acoustic path reference; weakening or disappearance of Rm reflects the transition from inter-sheet contact to metallurgical bonding; the degree of Rd bending is related to welding thermal-cycle intensity; and the evolution of Lu/Ld spacing is associated with through-thickness changes during nugget formation, growth, and solidification. M-type images therefore transform scattered instantaneous A-scan echo changes into continuous feature streaks that support physical interpretation, providing the basis for comparing M-type image features under different heat inputs and analyzing their correlations with nugget geometry.

### 3.3. M-Type Image Features Under Different Heat Inputs and Nugget States

Welding heat input determines nugget formation state and final geometry. Under different heat inputs, the interface echo, back-wall echo, and solid–liquid-interface-related echoes in M-type images evolve differently [11]. To establish the relationship between in-process ultrasonic features and nugget diameter D and thickness H, four typical states were selected based on metallographic morphology and measurements: under-welded/no-nugget, small-nugget, normal-nugget, and large-nugget conditions. For each state, the M-type image and corresponding metallographic image are presented together to compare process echo features with final nugget morphology.

**Figure 9:**
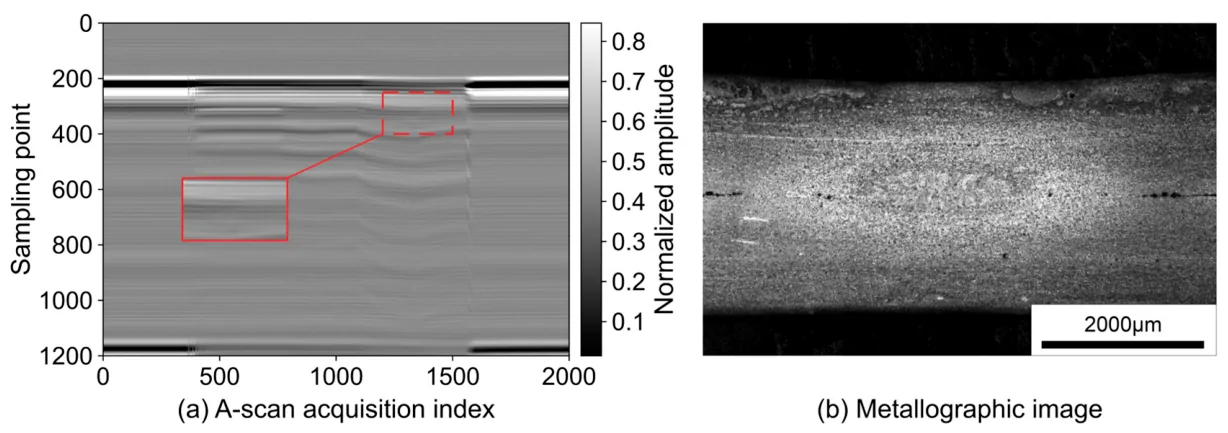
Comparison between the M-type image and metallographic morphology under the under-welded/no-nugget condition: (**a**) M-type image and (**b**) metallographic morphology. Red dashed outlines mark the regions of interest; the lower-left insets show enlarged views.

Figure 9 corresponds to the under-welded or no-nugget condition caused by severely insufficient heat input. No obvious nugget is formed in the metallographic image. In the M-type image, stable Lu/Ld responses are absent, and the middle region is dominated by the Rm streak from the inter-sheet interface, with no obvious broadening or redistribution during pulse II. The limited Rd bending suggests that heat input causes only a small change in sound velocity. Thus, the absence of the Lu/Ld-related response can provide a qualitative acoustic reference for assessing whether a nugget has formed effectively.

**Figure 10:**
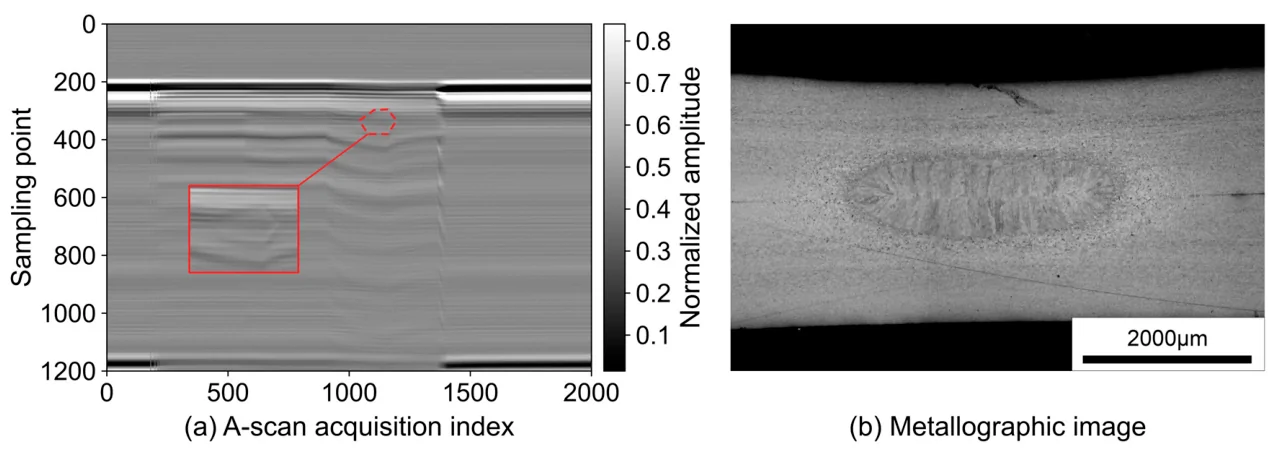
Comparison between the M-type image and metallographic morphology under the small-nugget condition: (**a**) M-type image and (**b**) metallographic morphology. Red dashed outlines mark the regions of interest; the lower-left insets show enlarged views.

Figure 10 corresponds to the small-nugget condition. The metallographic image shows a small nugget, and the M-type image shows local Lu/Ld-related responses with limited process-direction continuity, weaker gray-level contrast than in the normal-nugget condition, and small spacing. This indicates that lower heat input has produced localized fusion bonding, but through-thickness nugget growth remains limited, and the solid–liquid-interface-related echoes have not yet formed clear and sufficiently expanded streaks. Rd bending increases slightly compared with the under-welded condition but remains smaller than that in the normal-nugget condition.

**Figure 11:**
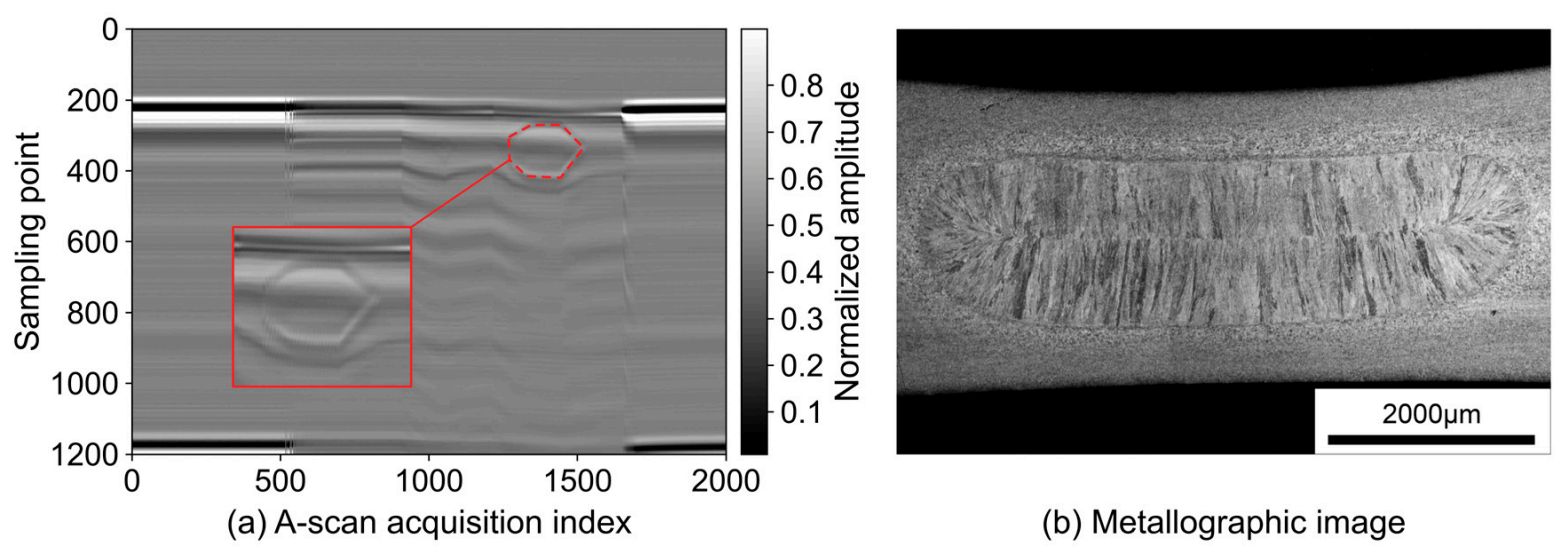
Comparison between the M-type image and metallographic morphology under the normal-nugget condition: (**a**) M-type image and (**b**) metallographic morphology. The red dashed outline marks the region of interest; the lower-left inset shows an enlarged view.

As heat input increases, the specimen shown in Figure 11 exhibits a relatively complete nugget morphology. In the M-type image, Rm gradually weakens, Lu/Ld responses clearly appear and broaden, and Rd bends markedly during pulse II. Compared with the under-welded and small-nugget conditions, the normal-nugget samples show clearer and more continuous Lu/Ld evolution during pulse II and cooling, with larger Lu/Ld spacing and a longer process-direction response than in the small-nugget condition.

**Figure 12:**
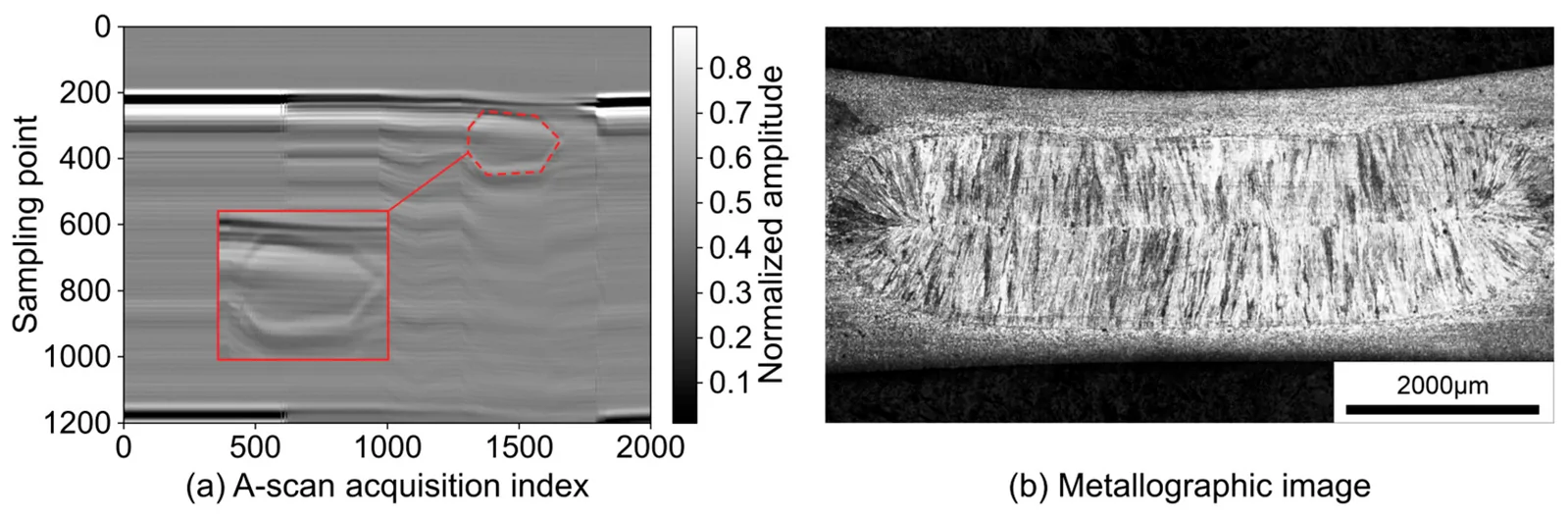
Comparison between the M-type image and metallographic morphology under the large-nugget condition: (**a**) M-type image and (**b**) metallographic morphology. The red dashed outline marks the region of interest; the lower-left inset shows an enlarged view.

Figure 12 corresponds to the large-nugget condition under higher heat input. The metallographic image shows a markedly larger nugget, with both D and H greater than those of the normal nugget. In the M-type image, Lu/Ld spacing further increases, response streaks cover a wider process-direction range, and local gray-level redistribution and streak-scale changes become more pronounced. Compared with the normal-nugget condition, the solid–liquid-interface-related responses persist longer and extend over a larger spatial range, and Rd bending is also stronger, reflecting the combined influence of increased nugget geometry and temperature-field variation on the ultrasonic propagation path and boundary reflection conditions.

The four representative samples show that the nugget state is mainly reflected in the continuity, range, spacing, and gray-level redistribution of Lu/Ld-related responses, together with the bending amplitude of the back-wall echo. Under-welded samples lack stable Lu/Ld responses; small-nugget samples show localized and discontinuous responses with small Lu/Ld spacing; normal-nugget samples show clear stage-dependent streaks and moderate spacing; and large-nugget samples show wider streak ranges and more pronounced gray-level redistribution. These signal differences involve echo intensity, spatial distribution, and changes between consecutively acquired signals, and are difficult to describe fully with a single peak value or acoustic path difference. Therefore, the subsequent quantitative evaluation uses the fixed statistical measures defined in Section 2.3 and Table 3 to characterize amplitude, energy, process-direction changes, and window peak-position relationships in the complete M-type matrix, with frequency-domain features providing complementary information on the frequency components of the waveforms.

### 3.4. Feature Fusion and Nugget Geometry Evaluation

The gray-level variations, streak extents, and process-direction changes observed in the preceding M-type images provided a basis for constructing numerical features. For quantitative analysis, the features listed in Table 3 were extracted from the complete two-dimensional A-scan matrices using the fixed windows and statistical rules described in Section 2.3. The manual echo annotations in the figures illustrate process phenomena. Manually traced trajectories were not included as model inputs. For D, regional peak amplitude, regional envelope energy, and overall envelope energy summarize gray-level intensity and response distribution in the M-type images. The process-direction gradient describes the mean change between consecutively acquired signals, and the high-frequency energy ratio provides complementary frequency-domain information on the relative power in the high-frequency band.

For H, the coefficient of variation in peak amplitude in window W_4_ describes relative fluctuations in the window peak amplitude along the acquisition direction. Normalized inter-window peak spacing and normalized peak position in window W_1_ describe relative peak-position relationships of the mean envelope curve within the fixed windows. The gray-level redistribution, streak changes, and relative echo-region positions observed in the images are thus summarized by numerical descriptors of amplitude, energy, gradient, and peak position. The high-frequency energy ratio additionally describes the frequency components of the waveforms. The fixed windows ensure consistent calculation rules across specimens and are not directly equated with a specific physical interface. This low-dimensional combination was used to retain statistical information on these signal differences and to reduce the risk of overfitting associated with excessive input dimensionality in a limited dataset [26].

The fixed feature combinations, random forest parameters, and LOOCV procedure described in Section 2.3 were used [24]. Separate prediction models were established for D and H. The quantitative relationships between these acoustic features and the metallographically measured D and H were evaluated using the 12 held-out predictions.

**Figure 13:**
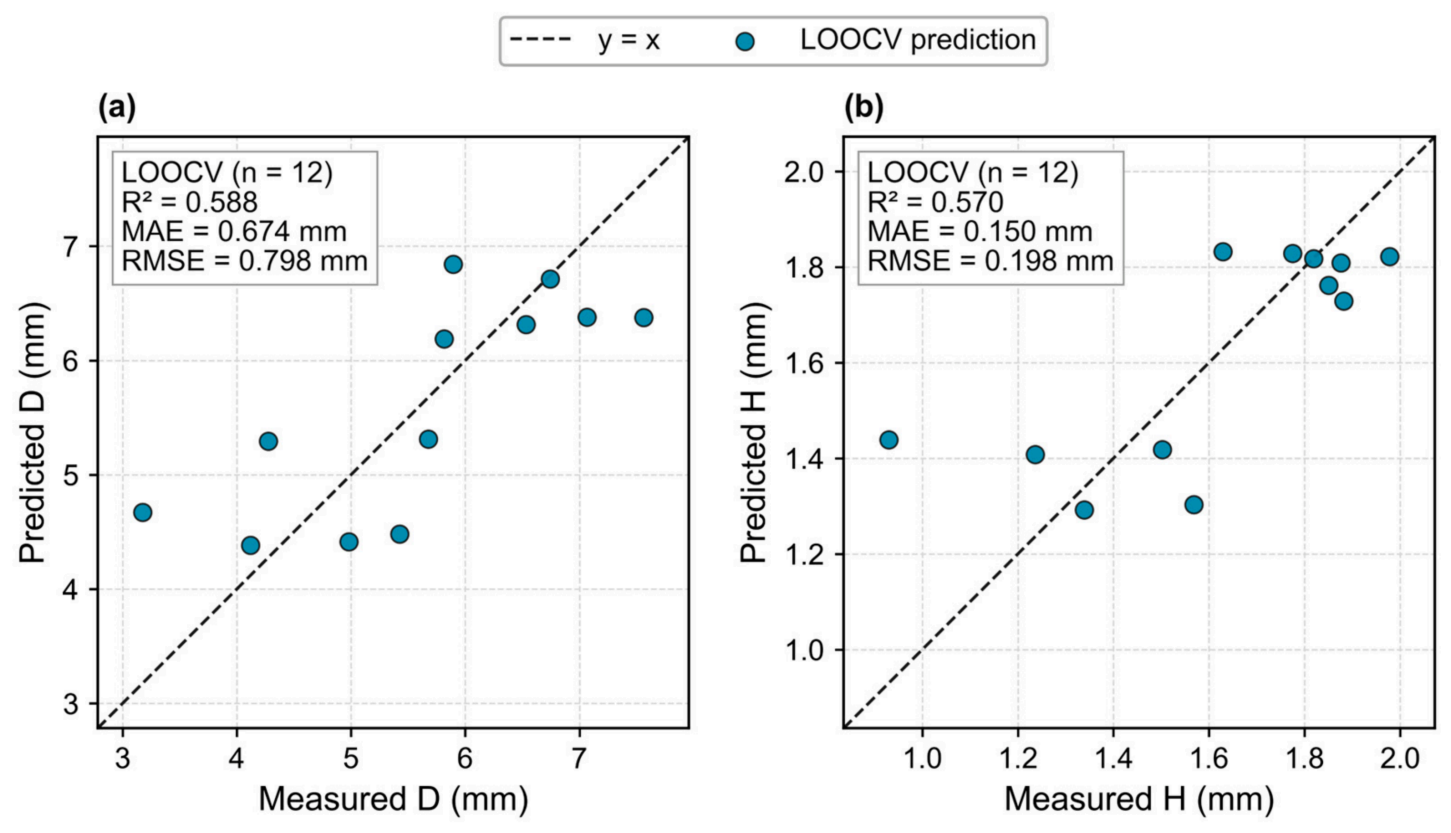
LOOCV prediction results for nugget diameter D and nugget thickness H: (**a**) nugget diameter D and (**b**) nugget thickness H.

Figure 13 shows the LOOCV out-of-fold prediction results for the 12 specimens. For nugget diameter D, R^2^ = 0.588, MAE = 0.674 mm, and RMSE = 0.798 mm, with a label range of approximately 3–7.5 mm. For nugget thickness H, R^2^ = 0.570, MAE = 0.150 mm, and RMSE = 0.198 mm, with a label range of approximately 0.9–2.0 mm. R^2^ was calculated from the 12 pooled held-out predictions and quantifies the reduction in the sum of squared residuals relative to a label-mean baseline. The two R^2^ values indicate that the fixed low-dimensional features can be used for preliminary prediction of nugget diameter and thickness. MAE represents the mean absolute error, whereas RMSE is more sensitive to larger deviations. Because D and H have different label ranges, their absolute errors should not be compared directly. The smaller MAE and RMSE for H do not indicate a higher coefficient of determination.

**Figure 14:**
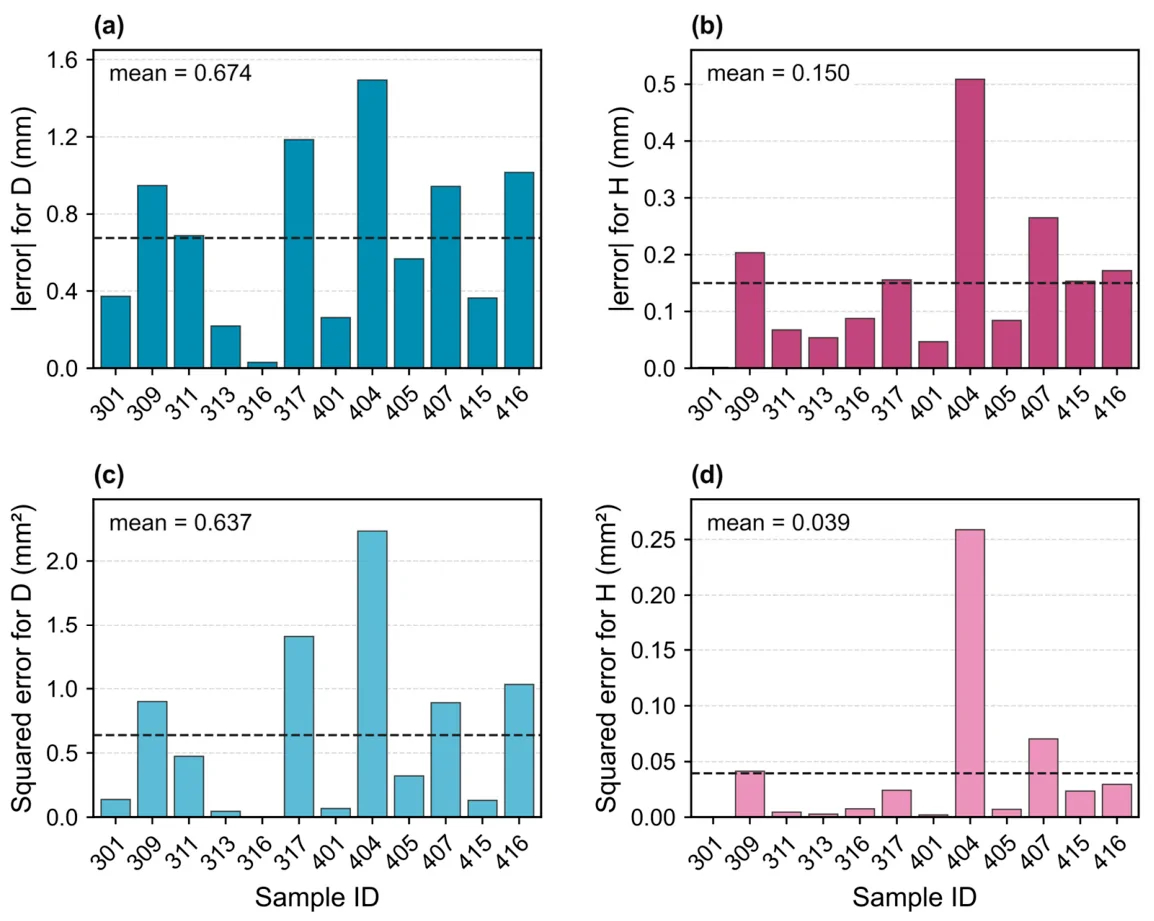
Sample-by-sample LOOCV error distribution: (**a**) absolute error for D, (**b**) absolute error for H, (**c**) squared error for D, and (**d**) squared error for H. The black horizontal dashed lines indicate the mean absolute errors in (**a**,**b**) and the mean squared errors in (**c**,**d**), calculated across all 12 specimens.

Figure 14 further shows the distributions of absolute error and squared-error contribution for the 12 leave-out samples. The errors for both D and H vary among samples, and a few samples make relatively large contributions to the total squared error, indicating sensitivity to individual samples. The representativeness of single-section metallographic measurements, nugget boundary determination, ultrasonic coupling state, temperature-related variations in sound velocity and attenuation, local echo quality, and model sensitivity under small-sample conditions may all affect prediction errors. Their individual effects were not quantified in this study.

Table 4 summarizes the LOOCV results for nugget diameter D and thickness H, indicating preliminary quantitative correlations between in-process ultrasonic features and nugget geometry. Given the limited sample size, the stability of predictive performance and generalization across welding conditions require further validation. Future work should increase the sample size, improve coverage of different welding conditions and nugget dimensions, and include repeat tests under the same welding conditions. It should also improve the consistency of metallographic measurements, optimize feature extraction and selection, and evaluate prediction accuracy and generalization using an independent test set [27].

## 4. Conclusions

(1) During the thermal cycle of dual-pulse resistance spot welding, the temperature field, inter-sheet contact state, and state of nugget formation varied across welding stages. In the A-scan signals, weakening of the original inter-sheet interface reflection and the appearance of echoes related to the nugget solid–liquid interface corresponded to the nugget formation stage. These acoustic responses can be used to identify changes in the welding process state.
(2) M-type images constructed from consecutive A-scan signals showed streak migration, broadening, bending, and gray-level redistribution, with distinguishable image responses for different nugget states. Based on echo amplitude, energy, process-direction changes, spectral distribution, and peak-position relationships, five-dimensional diameter features and three-dimensional thickness features were constructed to link qualitative observations of process echoes with quantitative analysis of nugget geometry.
(3) LOOCV evaluation was performed using fixed low-dimensional features and RF models for 12 specimens with metallographic measurements of D and H. For D, R^2^, MAE, and RMSE were 0.588, 0.674 mm, and 0.798 mm, respectively; the corresponding values for H were 0.570, 0.150 mm, and 0.198 mm. These preliminary results indicate a quantitative relationship between the extracted low-dimensional acoustic features and nugget diameter and thickness, providing experimental evidence for nugget-geometry evaluation based on in-process ultrasonic signals.

## Figures and Tables

**Table 1:** Main chemical composition of austenitic stainless steel (wt.%).

Element	C	Mn	Si	P	S	Ni	Cr	N
**Content**	**≤0.03**	**≤2.00**	**≤1.00**	**≤0.045**	**≤0.03**	**6.00–8.00**	**16.00–18.00**	**≤0.20**

**Table 2:** Acoustic constants of austenitic stainless steel.

Density/(kg·m^−3^)	Longitudinal Wave Velocity/(m·s^−1^)	Poisson’s Ratio (Dimensionless)	Wavelength at 10 MHz/mm
7930	5900	0.29	0.59

**Table 3:** Definitions of acoustic features used to predict nugget diameter D and thickness H.

Feature (Prediction Target)	Formula	Signal Quantity Represented
High-frequency energy ratio (D)	Σ_f≥0.25_ P(f)/[Σ_f_ P(f) + ε]	Relative power in the high-frequency band
Process-direction gradient (D)	mean_i,z_ |I(i + 1,z) − I(i,z)|	Mean change between consecutively acquired signals
Regional peak amplitude (D)	max_z∈W2_ p(z)	Peak of the mean envelope curve in window W_2_
Regional envelope energy (D)	mean_i,z∈W2_ E(i,z)^2^	Mean squared envelope in window W_2_
Overall envelope energy (D)	mean_i,z_ E(i,z)^2^	Mean squared envelope over the entire matrix
Coefficient of variation in peak amplitude in window W_4_ (H)	σ_b_/(μ_b_ + ε)	Relative amplitude fluctuation along the process direction
Normalized inter-window peak spacing (H)	max[0, (k_3_ − k_2_)/(N_z_ − 1)]	Spacing between mean envelope peak positions in windows W_2_ and W_3_
Normalized peak position in window W_1_ (H)	k_1_/(N_z_ − 1)	Peak position of the mean envelope curve in window W_1_

**Table 4:** LOOCV results for preliminary nugget-geometry evaluation using fixed acoustic features.

Geometric Endpoint	Feature Set	Model	Samples	*R* ^2^	MAE/mm	RMSE/mm
Nugget diameter	Five-dimensional diameter features	Random forest	12	0.588	0.674	0.798
Nugget thickness	Three-dimensional thickness features	Random forest	12	0.570	0.150	0.198

## Data Availability

The original contributions presented in this study are included in the article/Supplementary Materials. Further inquiries can be directed to the corresponding author.

## Supplementary Materials

The following supporting information can be downloaded at: https://www.mdpi.com/article/10.3390/ma19184001/s1, Table S1: Welding conditions and specimen counts represented by saved ultrasonic records.
