# Supplementary material for: In-Process Ultrasonic Monitoring and Nugget Geometry Evaluation in Resistance Spot Welding of Stainless Steel
Source: Materials (Basel). 2026 Sep 20;19(18):4001. doi: 10.3390/ma19184001 (PMC13608464; doi:10.3390/ma19184001)
Supplement: Supplementary file 1 [file materials-19-04001-s001.zip › materials-4550894-supplementary.pdf]

Supplementary Materials

In-process ultrasonic monitoring and nugget geometry evaluation in resistance spot welding of stainless steel

Manuscript ID: materials-4550894

Table S1. Welding conditions and specimen counts represented by saved ultrasonic records.

Condition	I_1/I_2 (kA)	$T_1/Tc/T_2$ (cycles)	Pressure setting (MPa)	Specimens with saved records	Included in D/H analysis
1	0/7.5	0/0/11	0.3	2	1
2	0/7.5	0/0/15	0.3	2	0
3	0/9	0/0/15	0.3	2	0
4	0/7.5	0/0/20	0.3	2	0
5	5/7.5	10/10/15	0.3	2	1
6	5/9	10/10/15	0.3	2	1
7	5/8	10/10/15	0.3	2	1
8	5/8.5	10/10/15	0.3	2	1
9	5/9.5	10/10/15	0.3	2	1
10	5/7.5	10/10/20	0.3	2	0
11	5/8.5	10/10/20	0.3	2	0
12	5/8	10/10/20	0.3	2	0
13	5/9	10/10/20	0.3	1	0
14	5/9.5	10/10/20	0.3	2	0
15	3/6	10/10/15	0.3	2	2
16	2/5	10/10/15	0.3	2	0
17	2/5.5	10/10/15	0.3	2	1
18	3/6.5	10/10/15	0.3	2	1
19	4/7	10/10/15	0.3	2	1
20	2/4	10/10/15	0.3	2	0
21	5/7.5	10/10/15	0.2	1	0
22	5/7.5	10/10/15	0.15	3	0
23	3/6	10/10/15	0.15	1	1
24	0/5	0/0/15	0.3	2	0
25	0/5.5	0/0/15	0.3	1	0
26	0/6	0/0/15	0.3	1	0
27	0/6.5	0/0/15	0.3	1	0
28	0/1.5	0/0/15	0.3	1	0
29	0/3	0/0/15	0.3	1	0
30	0/7.5	0/0/15	0.15	2	0
**Total**	—	—	—	**53**	**12**

Note: I_1 and I_2 denote the first- and second-pulse currents. T_1, Tc, and T_2 denote the first-pulse duration, inter-pulse cooling interval, and second-pulse duration. For single-pulse conditions, current and duration are listed under I_2 and T_2; I_1, T_1, and Tc are zero. The saved-record count refers to specimens represented by retained ultrasonic records, not a confirmed total of all specimens fabricated or tested. The final column identifies the 12-specimen D/H subset, covering 11 conditions. A zero means no specimen from that condition was included in this subset, not that no ultrasonic record was saved.
